# Progesterone Provides the Pleiotropic Neuroprotective Effect on Traumatic Brain Injury Through the Nrf2/ARE Signaling Pathway

**DOI:** 10.1007/s12028-016-0342-y

**Published:** 2016-12-19

**Authors:** Mei Zhang, Jianyue Wu, Haojun Ding, Wentian Wu, Guomin Xiao

**Affiliations:** Department of Neurosurgery, Affiliated Hospital of Hangzhou Normal University, Hangzhou, 310015 China

**Keywords:** Progesterone, Nrf2/ARE signaling pathway, Traumatic brain injury, Neuroprotection, Nrf2 gene knockout, In vivo

## Abstract

**Objective:**

This study was to investigate the role of Nrf2/ARE signaling pathway in the pleiotropic neuroprotective effect of progesterone (PROG) on traumatic brain injury (TBI).

**Methods:**

The Nrf2-knockout (Nrf2−/−) and C57 mice were respectively subjected to a lateral cortical impact injury caused by a free-falling object and randomly divided into three groups: sham-operated, trauma, and trauma + PROG treatment group. The PROG treatment group was given PROG (32 mg/kg of body weight, intraperitoneal injection) immediately after injury. For all groups, a series of brain samples were obtained after trauma at 24 and 72 h, respectively. The cerebral edema was evaluated; the expression of IL-1β, IL-6, and TNF-α was measured using ELISA array, and the apoptosis index was detected by TUNEL. Flow cytometry was used to detect the intracellular calcium concentration.

**Results:**

The water content, the apoptosis index, the levels of inflammatory cytokine, and the intracellular calcium ion were significantly decreased with the PROG treatment in C57 mice with TBI model. However, the effect of PROG on TBI was not found in the Nrf2−/− mouse model of TBI.

**Conclusions:**

PROG reduced cerebral edema, apoptosis, inflammatory reaction, and intracellular calcium ion overload effects after TBI. These beneficial effects were not seen in the Nrf2−/− mouse model of TBI. The results from this study suggested that the Nrf2/ARE signal pathway may be involved in the pleiotropic neuroprotective effect of PROG on TBI.

Traumatic brain injury (TBI) is a leading cause of morbidity and mortality in young adults, resulting in large direct and indirect social loss [1]. TBI is caused by both primary injury due to the initial biomechanical effects force on brain tissues and subsequent secondary injury due to activation of several pathophysiologic cascades. Brain damage and dysfunction after TBI have been attributed to a large extent to the secondary neurological injury mechanisms. It is recognized that the pathophysiology of secondary brain injury is a complex cascade of molecular and biochemical events after the onset of insult, such as neuronal excitotoxicity [2, 3], intracellular Ca^2+^ influx [4], free radicals, and lipid peroxidation [5, 6], exacerbated inflammatory response [7, 8], or subsequent neuronal cell death via necrosis and apoptosis [9, 10].

Research has shown that progesterone (PROG) has substantial pleiotropic properties as a neuroprotective agent in both animal models and humans [11, 12]. PROG attenuates glutamate excitotoxicity [13], modulates apoptotic pathways [14], reduces membrane lipid peroxidation [15], and limits inflammation after TBI [16]. PROG is synthesized by oligodendrocytes and some neurons in the brains of both sexes [17, 18]. PROG receptor is widely expressed in the central nervous system, so various brain regions are the normal targets of PROG [19]. PROG can also elicit its neuroprotective effects via non-genomic mechanisms such as through the activation of signal transduction pathways. Those second signal transduction systems are known to be activated by PROG include cAMP/PKA [20], MAPK (ERK1/2) [21, 22], TLRs/NF-κB [23], and the PI-3K/Akt signaling pathway [17]. Activation of such signaling pathways indicates its neuroprotective effects on brain injury. However, the various pathways that mediate simultaneously the pleiotropic neuroprotective effect of PROG on TBI are still unclear.

Nuclear factor erythroid-2p45-related factor 2 (Nrf2) is the family of transcription factors and binds antioxidant response element (ARE) in the promoter regions of antioxidant genes and phase II detoxification enzymes, which has been reported to be a pleiotropic regulator in the cellular defense mechanisms [24, 25]. Under basal conditions, Nrf2 is localized within the cytoplasm by binding to the cytosolic regulatory protein Keap1. Following activation, Nrf2 translocates to the nucleus where it binds to ARE, and enables gene transcription [26].

Despite the demonstrated role of PROG in neuroprotection on TBI, none of the previous studies focused on the Nrf2/ARE signaling pathway in relation to TBI. We hypothesized that the effect of PROG on activating the Nrf2/ARE signaling pathway was the mechanism whereby PROG provides the pleiotropic neuroprotective effect on TBI. The aim of this study was to investigate whether PROG activates Nrf2/ARE signaling pathway in a mouse model of TBI.

## Methods

### Animals

A total of 36 C57 mice (obtained from Hangzhou Normal University, Hangzhou, China), weighing 25 ± 2 g, were housed four to a cage in air-filtered, temperature-controlled units with 12-h light/dark cycle and regular food and water supply. Breeding pairs of 36 Nrf2−-deficient ICR mice were obtained from Nanjing Qingzhilan Techonolgy Co., Ltd (Nanjing, China). Homozygous wild-type Nrf2+/+ mice and Nrf2−/−-deficient mice were generated from inbred heterozygous Nrf2+/− mice [27]. Genotypes of Nrf2−/− and Nrf2+/+ mice were confirmed by polymerase chain reaction amplification of genomic DNA isolated from the blood. PCR amplification was performed using three different primers, 5′-TGGACGGGACTATTGAAGGCTG-3′ (sense for both genotypes), 5′-CGCCTTTTCAGTAGATGGAGG-3′ (antisense for wild-type), and 5′-GCGGATTGACCGTAATGGGATAGG-3′ (antisense for LacZ). The study protocol was approved by the Committee of Animal Care and Use for Research and Education (CACURE) of the Affiliated Hospital of Hangzhou Normal University and complied with the National Institutes of Health (NIH) guidelines for the care and use of laboratory animals.

### Models of Cortical Contusion Trauma

All mice were anesthetized with pentobarbital sodium (60 mg/kg) through intraperitoneal injection and fixed on a stereotaxic instrument. The skin of the right hemisphere was opened to expose the skull, a hole was drilled into the skull at the level of the parietal cortex (2 mm to the lambda, 2 mm to the fontanel, and 2 mm to the sagittal suture), and the exposed dura was kept intact. Trauma was induced by a modification of the Feeney’s weight-drop model [28] in which a free-falling weight drops onto the exposed intact cranial dura to produce a standardized parietal contusion (weighing 20 g, falling from 12 cm height). After trauma, the skull hole is closed with bone wax and the scalp was sutured. The sham-operated mice underwent the same surgical procedure without being exposed to percussion injury.

### Experimental Protocol

The mice were randomly assigned to three groups: (1) sham-operated group (sham); (2) trauma group (TBI); and (3) trauma + PROG treatment group (TBI + PROG). PROG (purchased from Xianju Pharmaceutical Co., Ltd, China) dissolved in 50 % dimethyl sulphoxide (10 mg/ml) and 50 % saline was immediately given as an intraperitoneal injection in TBI + PROG group after trauma (32 mg/kg body weight). Both sham and TBI groups received the same volume/weight of vehicle injection only. The mice were killed by decapitation at 24 and 72 h, respectively, after injury.

### Brain Tissue Water Content Measurement

A part of the mice brain (about 5 mm × 5 mm × 5 mm tissue cubes) was excised from the parietal cortex directly adjacent to the lesion core. The wet weight (WW) was rapidly measured with a chemical balance. The tissue was then dried in a desiccator oven at 100 °C for 24 h to reach constant dry weight (DW). The tissue water content was calculated as [(WW − DW)/WW] × 100 %.

### TUNEL Labeling

The sample cortex surrounding the contusion area or surrounding the parietal craniotomy in the sham group was collected. Formalin-fixed tissue was embedded in paraffin and sectioned at 4 μm with a microtome. The paraffin-embedded sections were also detected for apoptotic cells by terminal deoxynucleotidyl transferase dUTP nick-end-labeling (TUNEL) staining. The procedures were performed according to the instructions provided with the kit (Nanjing KaiJi Technology Development Co., Ltd). Paraffin sections of 5 μm thick were deparaffinized, and endogenous peroxidase was blocked by immersing the sections in phosphate-buffered saline (PBS) containing 0.5 % H_2_O_2_ for 15 min. For proteins nuclei stripping, tissue sections were incubated with proteinase K (20 μg/ml) for 10 min. The sections were incubated with a mixture of terminal deoxynucleotidyl transferase (TdT) and reaction buffer containing digoxigenin-labeled dUTP. Hematoxylin staining was performed followed by incubation with anti-digoxigenin antibody conjugated with peroxidase. Positive cells containing the fragmented nuclear chromatin which is a characteristic of apoptosis have brown-stained nuclei. The extent of brain damage was evaluated by the apoptotic index (AI), which was the average number of TUNEL-positive cells in each section counted in 10 microscopic fields (at 400× magnification).

### Multiplex Cytokine Enzyme-Linked Immunosorbent Assay

A 3-mm coronal section was taken from the injured area over the parietal cortex, accurately weighed, snap-frozen in liquid nitrogen, and stored at −70 °C. Frozen brain samples were homogenized in 1 mL of buffer containing 1 mmol/L of PMSF, 1 mg/L of pepstatin A, 1 mg/L of aprotinin, and 1 mg/L of leupeptin in PBS solution (pH 7.2) with a glass homogenizer and then centrifuged at 12,000*g* for 20 min at 4 °C. The supernatant was then collected, and total protein was determined by the Bradford method. The levels of inflammatory cytokines were quantified using enzyme-linked immunosorbent assay (ELISA) kits specific for rats according to the manufacturers’ instructions (Uscnk, USA). The cytokine contents in the brain samples were expressed as picograms of antigen per milligram of protein.

### Measurement of Intracellular Free Calcium ([Ca^2+^]_*i*_)by Flow Cytometry

The brain samples were washed twice with PBS, detached with a 0.125 % trypsin treatment (37 °C, 15 min), and further washed three times with PBS. For [Ca^2+^]_*i*_ detection, cell suspensions were incubated with fura-4-acetoxymethyl ester (final concentration 5 μmol/L, Gibco, USA) in the darkness at 37 °C for 40 min. Then cell suspensions were centrifuged at 1500 r/min for 5 min. After washing once in 2 mL PBS, the cells were resuspended in 0.5 mL PBS. The fluorescence of 10 000 cells was analyzed by flow cytometry (488 nm). The relative fluorescence intensity of Fluo-1 was used as the indication of [Ca^2+^]_*i*_ quantity.

### Statistical Analysis

All data were presented as mean ± standard deviation. SPSS 12.0 (SPSS Inc., Chicago, IL) was used for statistical analysis of the data. For evaluation data, repeated-measures analyses of variance (two-way or one-way as appropriate) and t tests were utilized to determine statistical differences. A Holm–Sidak method for multiple comparisons post hoc test was used to determine data points with significant differences. Statistical significance was inferred at *P* < 0.05.

## Results

### Brain Tissue Water Content Measurement

**Fig. 1:**
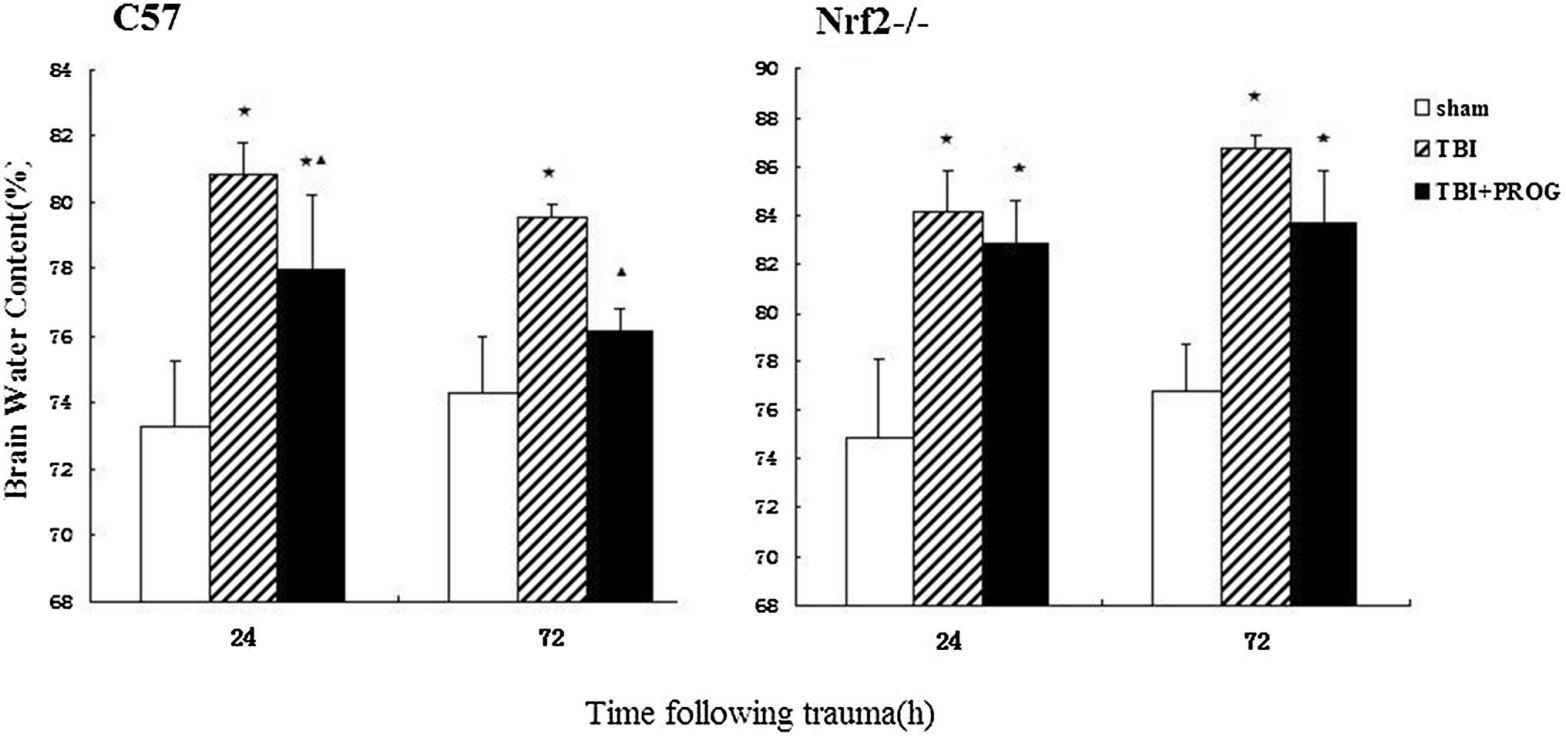
Alterations in brain tissue water content adjacent to the lesion site in sham group, TBI group, and TBI + PROG group (*n* = 12) between the C57 mice and Nrf2−/− mouse model of brain injury at 24 and 72 h after injury. The *right-hand* side illustrates the Nrf2−/− mouse model of TBI. The *left-hand* side is the C57 mouse model of TBI. The brain tissue water content is given as the mean ± SE. *P < 0.01 versus sham control and ^▲^ *P* < 0.05 versus TBI vehicle-treated injured mice

As shown in Fig. 1, in C57 mice with acute brain injury model, brain tissue water content adjacent to the lesion site in the injured group was significantly increased when compared to the sham group at 24 and 72 h after trauma, while tissue water content was not increased in PROG treatment group when compared with the sham group at each time points after trauma. Tissue water content in the TBI + PROG group was significantly reduced compared to the injured group at the same time after trauma. However, in the Nrf2−/− mouse model of brain injury, there was no statistical significance between the TBI + PROG treatment group and the trauma group with the water content of brain at 24 and 72 h after injury. PROG post-injury administration did not attenuate cerebral edema after trauma in the Nrf2−/− mouse model of TBI.

### Apoptotic Index Measured by TUNEL Labeling

**Fig. 2:**
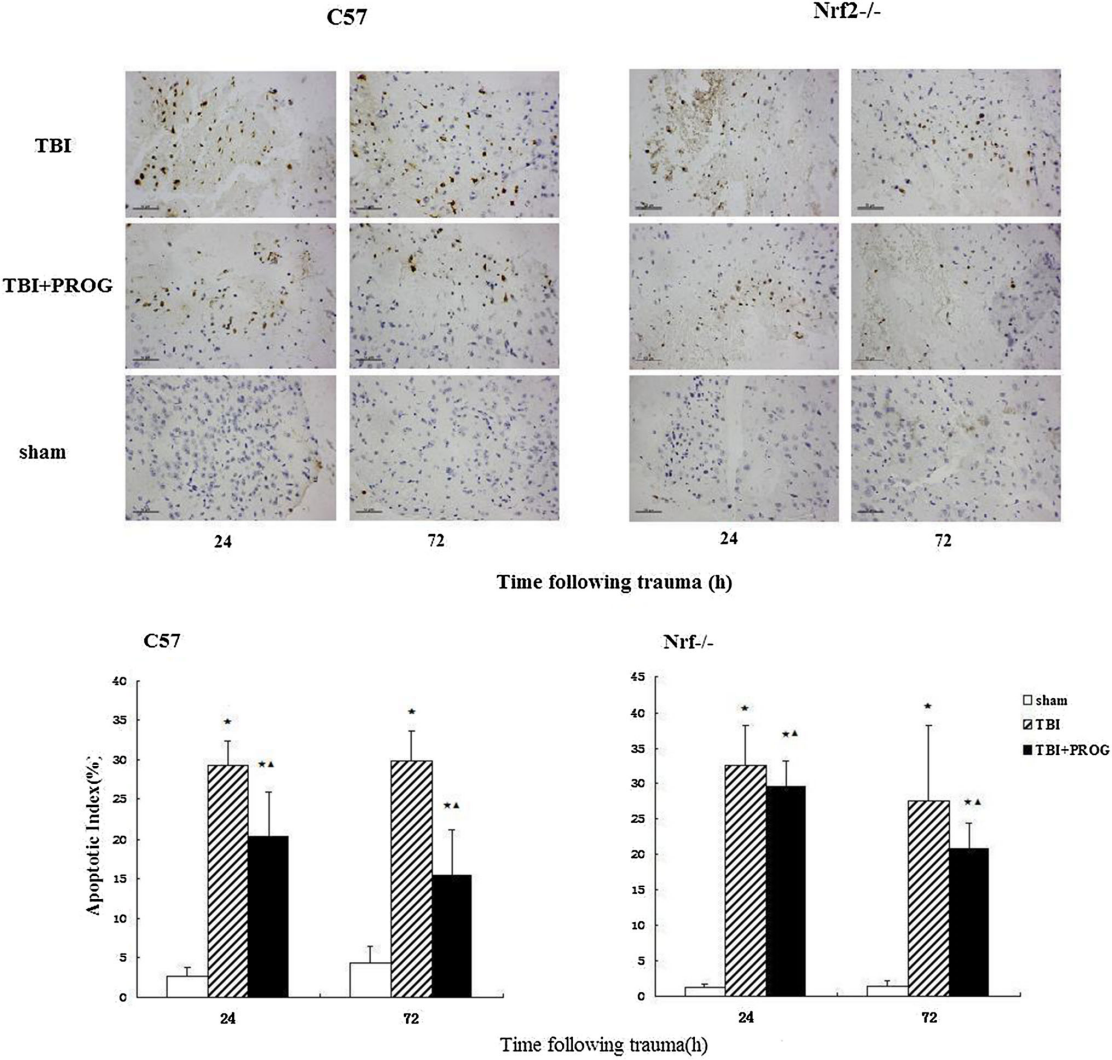
TUNEL staining of (×400) of apoptotic neurons in the cortex surrounding the injured site in sham group, TBI group, and TBI + PROG group (*n* = 12) between the C57 mice and Nrf2−/− mouse model of brain injury at 24 and 72 h after injury. The *right-hand* side illustrates the Nrf2−/− mouse model of TBI. The *left-hand* side is the C57 mice model of TBI. *Scale bars*, 50 μm. The apoptotic index is given as the mean ± SE. *P < 0.01 versus sham control and ^▲^ *P* < 0.01 versus TBI vehicle-treated injured mice

The number of apoptotic profiles was measured by TUNEL staining. Similar to the brain tissue water content measurement, PROG post-injury administration did not reduce nerve cell death after trauma in the Nrf2−/− mouse model of TBI. As shown in Fig. 2, in the C57 mouse model of TBI, few TUNEL-positive apoptotic cells were found in brains of the sham group. The apoptotic index in the cortex surrounding the injured site was found to be significantly increased compared to the sham group animals at 24 and 72 h after trauma in the TBI group. In TBI + PROG group, when compared to the TBI group, the apoptotic index in the cortex surrounding the injured site was significantly decreased. However, in the Nrf2−/− mouse model of TBI, there was no statistical significance between the TBI + PROG treatment group and the TBI group with the apoptotic index in the cortex surrounding the injured site at 24 and 72 h after injury. These results indicated that PROG administration following TBI leads to less cell death in Nrf2+/+ mice but not in mice lacking the Nrf2 gene.

### Multiplex Cytokine levels Quantified with ELISA Array

**Fig. 3:**
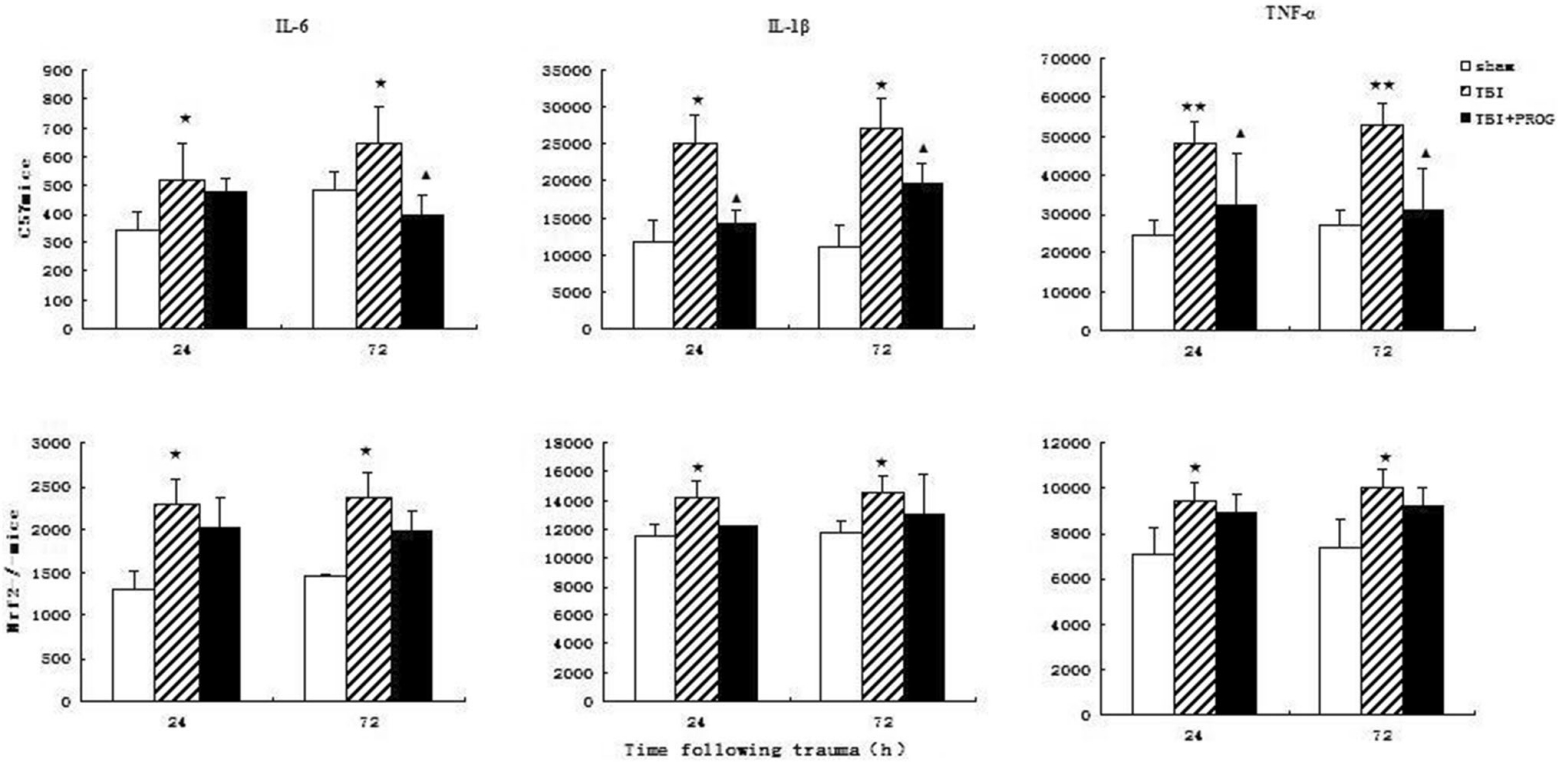
Changes of inflammatory mediators in sham group, TBI group, and TBI + PROG group (*n* = 12) between the C57 mice and Nrf2−/− mouse model of brain injury at 24 and 72 h after injury. The cytokine levels are given as the mean ± SE. *P < 0.05 and **P < 0.01 versus sham control and ^▲^ *P* < 0.05 versus TBI vehicle-treated injured mice

Levels of cytokines IL-1β, IL-6, and TNF-α in brain tissue lysate at 24 and 72 h after injury measured using a commercially available ELISA array are shown in Fig. 3. In C57 mice with TBI model, the inflammatory cytokine(IL-1β, IL-6, and TNF-α) levels in sham mice brain tissue at each time point after injury were consistently presented in a low background. All three measured cytokine levels exhibited significant increases in different time points. PROG treatment did produce significant reductions in the injury-induced up-regulation of IL-1β, IL-6, and TNF-α expression. However, in the Nrf2−/− mouse model of TBI, PROG treatment did not reduce IL-1β, IL-6, and TNF-α expression in brain tissues at 24 and 72 h after injury compared to the trauma group animals.

### [Ca^2+^]_*i*_ Measured by Flow Cytometry

**Fig. 4:**
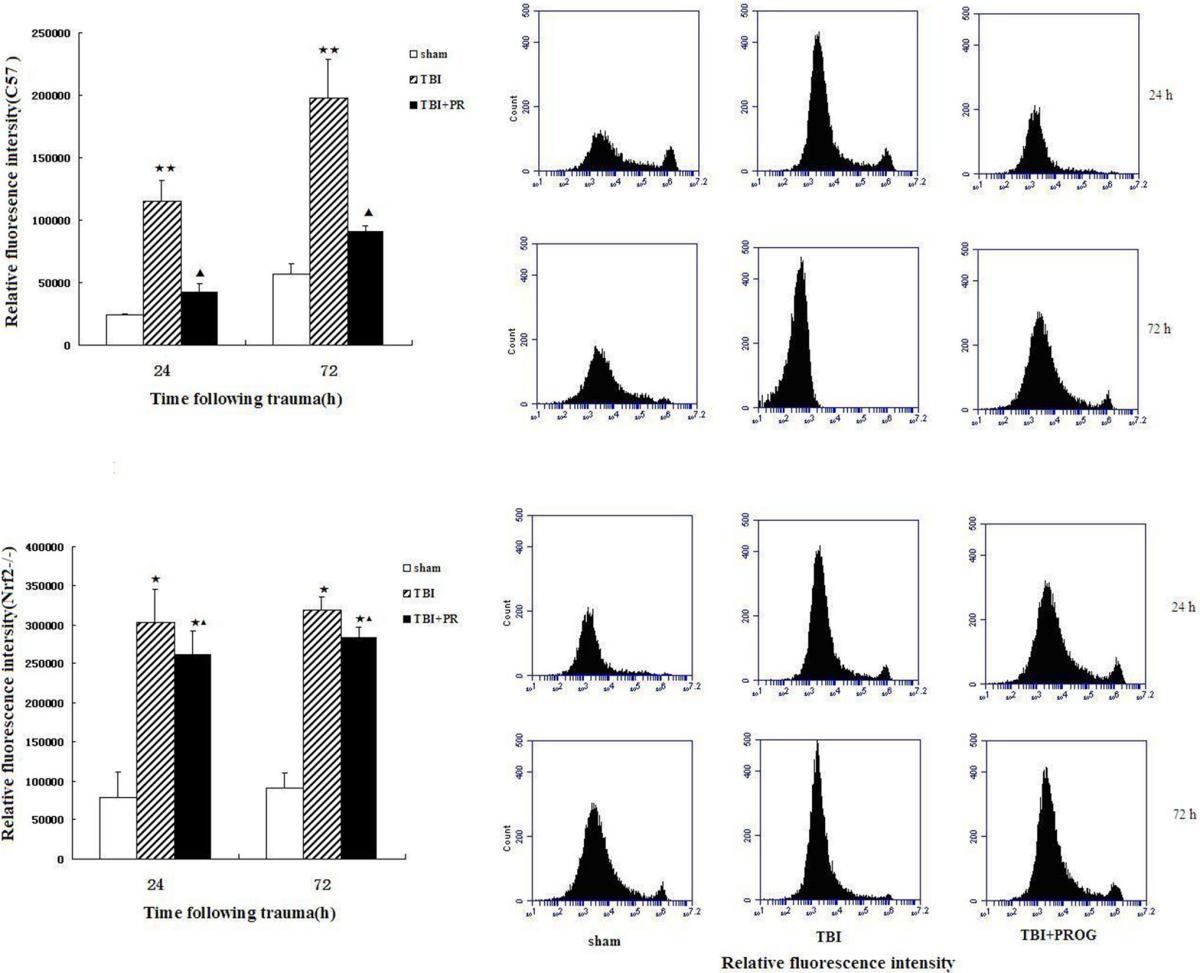
Relative fluorescence intensity of intracellular free calcium in sham group, TBI group, and TBI + PROG group (*n* = 12) in the C57 mice and Nrf2−/− mouse model of brain injury at 24 and 72 h after trauma. The *right-hand* side illustrates the flow cytometric analysis of brains in sham group, TBI group, and TBI + PROG group of C57 mice and Nrf2−/− mice brain tissues at 24 and 72 h after injury. The *left-hand* side is the quantitative presentation of the relative fluorescence intensity of intracellular free calcium. The *upper line* is the C57 mouse model of TBI. The lower line is the Nrf2−/− mouse model of TBI. The relative fluorescence intensity of intracellular free calcium is given as the mean ± SE. *P < 0.05 and **P < 0.01 versus sham control and ^▲^ *P* < 0.05 versus TBI vehicle-treated injured mice

As shown in Fig. 4, the flow cytometric analysis showed a marked increase of the relative fluorescence intensity of [Ca^2+^]_*i*_ in the TBI group of C57 mice brain tissues at 24 and 72 h after injury. Compared to the TBI group, the relative fluorescence intensity of [Ca^2+^]_*i*_ in the TBI + PROG group at the same time points after injury was significantly reduced. However, in the Nrf2−/− mouse model of TBI, there was no statistical significance between the TBI + PROG treatment group and the trauma group with the relative fluorescence intensity of [Ca^2+^]_*i*_ in brain at 24 and 72 h after injury. Blockade of TBI-induced [Ca^2+^]_*i*_ by PROG in the C57 mice with TBI model was not observed in the Nrf2−/− mouse model of brain injury.

## Discussion

This study examined the role of Nrf2/ARE signaling pathway in the pleiotropic neuroprotective effect of PROG on TBI. We found that PROG has neuroprotective properties in C57 mice with acute brain injury model. Clearly, the mechanism of PROG in neuroprotection does not target a single aspect of the secondary injury cascade following TBI, but it instead works through multiple mechanisms to enhance neural repair caused by head injury; as such, PROG reduced cerebral edema, apoptosis, inflammatory reaction, and intracellular calcium ion overload effects after TBI. However, in the Nrf2-knockout mice model, our results showed that the loss of Nrf2 in vivo lessens the pleiotropic protective effects of PROG on TBI, consistent with a specific role for Nrf2/ARE signaling pathway. Collectively, we speculated that the pleiotropic neuroprotective effect of PROG on TBI is related to the Nrf2/ARE signal pathway. The results from our study confirm and extend these previous observations and demonstrate for the first time that post-injury administration of PROG may activate the Nrf2/ARE signal pathway, and this may be the mechanism underlying the pleiotropic neuroprotective effect of PROG on TBI.

Recently, two large, multicenter, randomized, double-blind, placebo-controlled phase III clinical trials evaluated the effectiveness of PROG in patients with TBI. The results from both trials revealed that application of PROG would not be beneficial in the treatment of TBI [29, 30]. Various factors have been proposed which may have contributed to the failure of TBI trials to demonstrate convincing efficacy in the TBI population. One major issue is the heterogeneous nature of TBI. Another reason may be that the neuroprotective mechanisms of PROG on TBI are still unclear. After extensive research on the treatment of PROG in TBI over the last three decades, it is clear that PROG is a neurosteroid that affects multiple mechanisms involved in neuroprotection and neural repair following various types of brain injury. These effects were associated with a reduction in edema [31] and reduce neurogenic inflammation [32]. PROG can upregulate the anti-apoptotic version of Bcl-2 and anti-apoptotic protein ERK [33, 34]. There is evidence that PROG can reduce lipid peroxidation [15] and attenuate neuronal excitotoxicity [13, 35, 36]. PROG can promote both central and peripheral remyelination through increasing myelin production [37–39]. In the current study, we found that PROG administration following TBI reduces apoptotic cell death and edema, and inhibits neurogenic inflammation and neuronal excitotoxicity in the brain tissue surrounding the cortical contusion, which was also observed by previous investigators.

PROG possesses pleiotropic neuroprotective effects that may markedly attenuate the injury cascade associated with TBI. However, the mechanism whereby PROG provides the effects on TBI is entirely unclear. Recently, the Nrf2/ARE signal pathway has been shown to play an indispensable role in attenuation multiple pathophysiological processes in brain injury, including oxidative stress, mitochondrial dysfunction, and inflammation. TBI can induce Nrf2/ARE pathway activation in the brain [40]. Furthermore, activation of the Nrf2/ARE pathway by a pharmacologic inducer is able to protect neurons in the animal models of Parkinson’s disease, cerebral ischemia, and intracerebral hemorrhage [41–43]. Specifically, the promising Nrf2/ARE activator sulforaphane has been demonstrated to protect neurons from TBI, including blood–brain-barrier dysfunction, brain edema formation, and cognitive deficits [44]. Another small molecule, carnosic acid, was shown to be neuroprotective in vivo in an animal model of cerebral ischemia. These protective effects of carnosic acid were also shown to be mediated by activation of the Nrf2/ARE pathway and hence through downstream mediators that are capable of eliciting a robust neuroprotective effect [45].

However, none of the previous studies have focused on the Nrf2/ARE signaling pathway in relation to the pleiotropic neuroprotection of PROG after TBI, despite the demonstrated role of PROG in neuroprotection. To further verify the causal relation between the Nrf2/ARE pathway activation by PROG and its pleiotropic neuroprotective effect in TBI, we used the TBI model of Nrf2-knockout mice. In the current study, we found that the pleiotropic neuroprotective effect of PROG on TBI reduced cerebral edema, apoptosis, inflammatory reaction, and intracellular calcium ion overload effects after TBI in Nrf2+/+ mice but not in mice lacking the Nrf2 gene, confirming both the neuroprotective action and the Nrf2 dependence of PROG in vivo. This study revealed that the Nrf2/ARE signaling pathway could be activated when treated with PROG. The findings reported here suggest for the first time that the Nrf2/ARE signal pathway may be involved in the pleiotropic neuroprotective effect of PROG on TBI.

In summary, to the best of our knowledge, this is the first study to demonstrate an effect of PROG on the Nrf2/ARE signaling pathway in the injured brain after TBI. The benefit of PROG administration after TBI may be due to its salutary effect on modulating the Nrf2/ARE signaling pathway. However, the study only presented preliminary evidence that PROG could exert pleiotropic neuroprotective effect against TBI via up-regulating the Nrf2/ARE signaling pathway. The present experimental results do not fully support this conclusion. Further experiments will be required to determine whether PROG exerts its pleiotropic neuroprotective effects via activation of Nrf2/ARE signaling pathways. This hypothesis could be tested by determining the upstream regulators of Nrf2/ARE, promoter binding sites, and the downstream effector genes of PROG.
